# AI-based prostate analysis system trained without human supervision to predict patient outcome from tissue samples

**DOI:** 10.1016/j.jpi.2022.100137

**Published:** 2022-09-08

**Authors:** Peter Walhagen, Ewert Bengtsson, Maximilian Lennartz, Guido Sauter, Christer Busch

**Affiliations:** a Spearpoint Analytics AB, Stockholm, Sweden; b Centre for Image Analysis, Dept. of Information technology, Uppsala University, Uppsala, Sweden; c Institute of Pathology, University Medical Center Hamburg-Eppendorf, Hamburg, Germany; d Dept. of Surgical Sciences, Uppsala University, Uppsala, Sweden

**Keywords:** Prostate cancer grading, Artificial intelligence-based cancer grading, Predicting prostate cancer recurrence

## Abstract

In order to plan the best treatment for prostate cancer patients, the aggressiveness of the tumor is graded based on visual assessment of tissue biopsies according to the Gleason scale. Recently, a number of AI models have been developed that can be trained to do this grading as well as human pathologists. But the accuracy of the AI grading will be limited by the accuracy of the subjective “ground truth” Gleason grades used for the training. We have trained an AI to predict patient outcome directly based on image analysis of a large biobank of tissue samples with known outcome without input of any human knowledge about cancer grading. The model has shown similar and in some cases better ability to predict patient outcome on an independent test-set than expert pathologists doing the conventional grading.

## Introduction

Prostate cancer is one of the most common cancers among men with around 1.4 million annual cases world-wide.^1^ The cancers vary widely in how aggressively they grow and it is important to grade the tumor to determine how the treatment should be to find the best balance between risk of deadly progression and effects of the treatment such as incontinence and erectile dysfunction. The established way of doing that grading is through inspection of tissue from biopsies according to a method introduced by Gleason.^2^^,^^3^ Unfortunately, the subjective evaluation of the Gleason patterns show substantial variation leading to over- or under-treatments.^4^^,^^5^ Recently, the Gleason system has been reformed to a different scale called ISUP but the problems with reproducibility and variability remains.^6^ Progress in AI development has led to a number of projects training AI systems to perform the Gleason grading, see for instance.^7^^,^^8^^,^^9^ A large challenge was organized in the spring of 2020 with more than 1000 participating groups^10^ resulting in a number of algorithms with good performance. While the developed AI models show impressive ability to produce grades with the same precision as experienced pathologists, their accuracies are limited by the subjective “ground truth” Gleason grades used for the training.

A potentially better approach would be to train the AI models on tissue from patients with prostate cancer for which the outcome is known. Based on a large biobank with prostate tissue samples from 17 700 patients with several years of follow-up data, we have been able to end-to-end train an AI model to predict which patients will have a relapse and which patients will live on with no further effects from their cancer. We trained our model to find patterns in the tissue that correlate to outcome without any subjective human input concerning where to look and what patterns to look for. To make the model clean cut, we only used a binary division of the patient outcome between those who did get a biochemical relapse, metastases, or death from cancer in one group and those who did not get any bad outcome for at least 5 years of follow up in the other group. We have called our model PCAI, Prostate Cancer Aggressiveness Index.

To the best of our knowledge, this is the first study showing ability of an AI system to be trained to predict prostate patient outcome completely without any subjective teaching. The most similar previous result is in a study by Dietrich et al^.^^11^ in which they were able to train a model to reach similar performance to our model based on training it on the same biobank. The approach included, however, an intermediate step where the model was trained to replicate Gleason grade, thus it is not obvious how much of the end result that is learned from the Gleason step. Our approach includes no human-biased data selection or teaching whatsoever, except ImageNet-pretrained weights which arguably are so out of domain that they merely provide well-tuned base features but no pathology domain-specific knowledge.

In the following sections, we describe the material used for our study (Material), the developed method (Method), the achieved results (Results), and a discussion and conclusion (Discussion and conclusion).

## Material

### The dataset

Our study was based on a large biobank of prostate tissue samples available at the Martini Klinik in Hamburg, Germany. The tissue samples were available in a tissue micro array, TMA, format providing one tissue sample of around 0.6 mm diameter for each case.^12^^,^^13^ The samples were stained with the conventional H&E stain. Each of the 39 TMAs contained between 129 and 522 tissue samples with an average of 454. A total of 17 700 patients with follow-up data and tissue samples were available for our study. The TMAs were scanned with an Aperio AT2 scanner using a 40X lens resulting in a pixel size of 0.25 micrometers.

The tissue samples in the dataset were sampled from representative cancer areas in radical prostatectomies, i.e. in prostates which had been surgically removed from the patients. Any biochemical recurrence of PSA after the surgery, thus with high probability comes from a metastasis as there has been no prostate left in the patient to produce the PSA. This makes biochemical recurrence (BCR) a strong signal which tells if the cancer was spread or not at the time of the tissue sampling. Further – local, but possibly cured during the surgery, spread was recorded. This included invaded and removed lymph nodes and extra-prostatic stage.

Table 1 shows an overview over the dataset used in the study.

**Table 1:** Data distribution of the dataset used in the study.

Feature	Number of data entries	Median value	Mean value
Number of cases total	17 700	–	–
Follow-up time total (until bad outcome or end of follow-up for other reasons)	15 905	4.1 years	5.3 years
Follow-up time (with no bad outcome)	12 143	4.0 years	5.0 years
With cancer-specific death	17 530	–	1.3 %
Time to cancer-specific death (of positive)	226	5.8 years	6.7 years
With BCR (PSA recurrence ≥ 0.2 ng/ml)	15 911	–	23.4 %
Time to BCR (of positive)	3716	1.7 years	2.6 years
With metastasis	11 933	–	5.3 %
Time to metastasis (of positive)	630	2.9 years	4.1 years
With bad outcome (Cancer-specific death OR BCR OR metastasis)	15 905	–	23.7 %
Time to bad outcome (of positive)	3762	1.8 years	2.6 years

pN (Lymph node metastasis)	11 900	–	10.5 %
pV, pL (vessel infiltration)	12 267	–	14.7 %
Local spread (pN OR pV OR pL)	14 913	–	15.9 %

Patient age	17 700	64.5 years	63.8 years
Preoperative PSA	17 608	6.9 ng/ml	10.0 ng/ml
Prostatectomy ISUP	17 682	2	2.3
pT (pathological stage)	17 690	2	2.3
Extraprostatic stage (pT ≥ 3)	6177	–	34.9 %

### Target space

The target space to train and evaluate the model against was chosen as a binary problem where patients were divided into 2 risk groups; those with signs of spread cancer (at the time of the tissue sample) and those with cancer most likely contained within the prostate.

The definition of the risk groups for training was:

*High risk*: Bad outcome within 3 years OR local spread.

*Low risk*: No bad outcome AND follow-up for more than 5 years AND no local spread.

And for testing:

*High risk*: Bad outcome within 3 years.

*Low risk*: No bad outcome AND follow-up for more than 5 years.

### Test sets

The dataset was split into a training set and a test set and then cleaned of spots with too low tissue content and those which could not define the target space.

To compare the model performance against human expert level 500 samples from the test set were annotated by a human expert uropathologist (Pathologist 1).

A second test dataset of 4181 samples from 828 patient cases, with follow-up data completely withheld from the development team, that had been annotated by 2 human expert uropathologists (Pathologist 1 and Pathologist 2) was provided. These samples were provided from separate TMA blocks to not make it possible to overfit the model against those sample’s TMA-specific parameters such as staining and thickness bias.

Table 2 shows the different test sets used in the study.

**Table 2:** The number of tissue samples used for our study.

Dataset	Number of samples	Fraction High risk	Fraction Low risk	Comment
Train	8826	66%	34%	
Test	1731	59%	41%	“Internal” test set. Drafted randomly from initial training dataset (not included in Train)
Test-CB	500	56%	44%	Subset of Test, annotated by Pathologist 1.
Test-UKEHDS	4181 (828 cases)	28% (BCR)	72% (BCR)	Separate test set annotated by Pathologist 1 and Pathologist 2.

## Method

### The PCAI Model

**Fig. 1:**
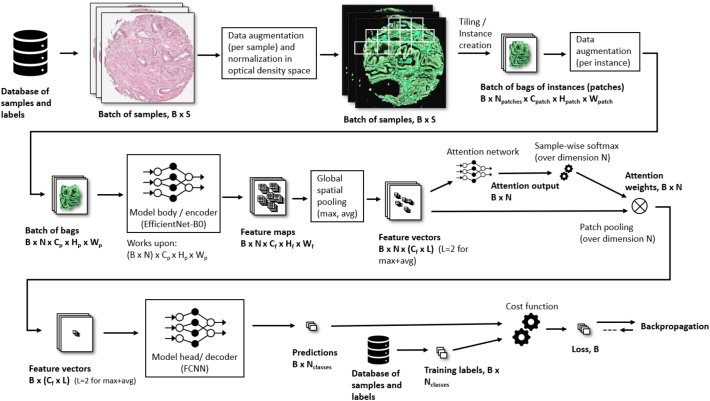
Model training pipeline.

Due to the large size of the images, ∼3000 × 3000 pixels, and to also prepare for the model to work on even larger image sizes from full-scale biopsies, the model was designed as an attention-based MIL^14^ model according to the pipeline in Fig. 1. Attention-based MIL allows an arbitrary number of instances together build up the sample which allows for flexible sampling schemes over tissue areas with different forms and sizes.

**Fig. 2:**
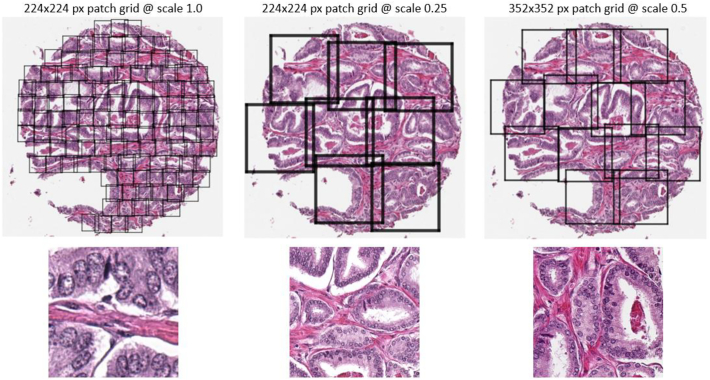
Grids for instance creation. Left: Grid laid out on scale 1.0, 224 px patches and an example patch. Mid: Scale 0.25, 224 px, Right: Scale 0.5 352 px.

Three separate models were trained with different settings in the instance creation stage to focus on different context of the tissue (see Fig. 2):

• Scaled 1.0 (40x), 224 px patches. This to give enough resolution (0.25 μm per pixel) to resolve nuclear chromatin texture.
• Scaled 0.25 (10x), 224 px patches. This to give enough receptive field (226 μm patch size) to reveal glandular architecture.
• Scaled 0.5 (20x), 352 px patches. This to give a bit of both of the above contexts.

These 3 models were then combined in a linear ensemble with weights 0.25, 0.25, 0.5 for the scale 1.0, 0.25, 0.5 models, respectively, to provide the final model (this weighing was optimized against the training set).

EfficientNet-B0^15^ was chosen as backbone for the encoder due to that it achieved best results in experiments and that it is a relatively small model, which leaves room for other things in the GPU memory, such as larger batch sizes, in the training. The cost function was cross-entropy as the problem setup was binary classification.

The attention network the role of which is to determine weights for how much to consider the different instances (patches) was designed as a 1 hidden layer fully connected neural network (FCNN) with 64 hidden neurons and mish activation^16^ and batchnorm.

The decoder network was a FCNN with 2 hidden layers of 768 resp 16 hidden neurons and mish activation + batchnorm.

### Data augmentation

Color information was randomly augmented per spot with perturbations (uniform distribution) to brightness (±2%), contrast (±5%), saturation (±20%), and hue (±5%) before the preprocessing. The grids to select patches were subject to random spatial shift and the patches were then randomly mirrored and/or rotated a multiple of 90 degrees resulting in 8 dihedral variants.

### Preprocessing

As preprocessing the images were normalized to zero mean and unit standard deviation in optical density space to focus on pigment content rather than light transmission.

*Image*_*optical density*_ *= -log(max(Image*_*linear*_ *, 1e* ^*-3*^ *))*

*Where Image*_*linear*_ *is in value range [0.0 1.0]*

**Fig. 3:**
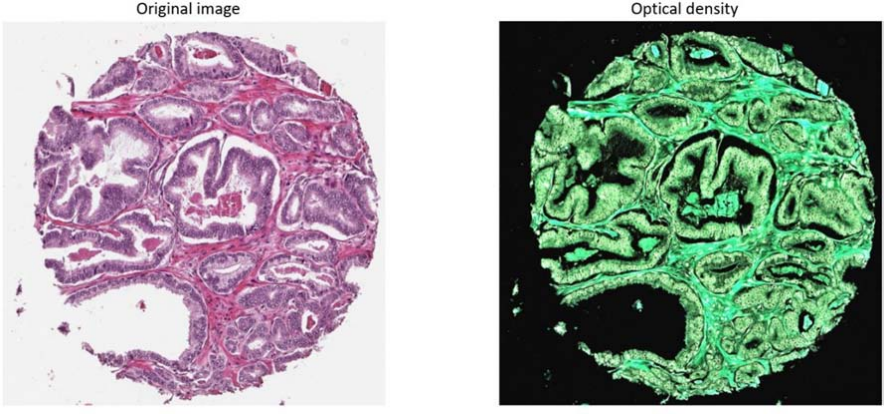
Left: Image in linear space. Right: Image in optical density space.

See Fig. 3 for a visualization of a tissue image in linear space and transformed to optical density space.

The rationale for using optical density is that it is a direct measure of the amount of stained, light absorbing tissue in each pixel.^17^ Also the interesting information lies in the low intensity range, i.e. where the H&E pigments are concentrated and light is absorbed. High intensity, like background, represents void of information. The logarithmic transform extends the dynamic range in the interesting low intensity region and squeezes the dynamic range in the uninteresting high intensity range and linear operations performed then in the optical density range will thus take more consideration to the interesting range than the uninteresting one.

Experiments showed that for a contained dataset the normalization in optical density space compared to normalization in linear space does not affect performance notably – but domain transfer (other scanners etc.) benefits from it.

### Training procedure

#### Denoising and pseudo-labeling

The training was performed in 2 stages. In stage 1, a prototype model was trained that was used to clean outlier data in the dataset (denoise) and include (pseudo label) some likely, but not confirmed due to too short follow-up time, low-risk samples. The final (stage 2) PCAI models were then trained on this curated dataset. Testing was performed both on the uncurated “raw” dataset and on the denoised “cleaned” dataset separately. The withheld test set was only tested in its “raw” setting as no follow-up info was available to the developer from this set.

The denoising was performed by discarding samples which had a prediction by the stage 1 model that disagreed a lot with the label.

The rationale for this cleansing is that as the TMA spots are so small, only 0.6 mm ⌀, there is a non-negligible risk for the spots being sampled at a non-representative location, i.e. the spot is unrepresentative of the whole prostate. A result illustrating this was obtained in the study of a small subset of all samples by Pathologist 1 described in section 4.3.

The risk with this kind of denoising is that “hard” cases, rather than unrepresentative samples are removed, but if more unrepresentative samples than hard ones are removed it should prove useful.

The pseudo-labeling was performed to increase the fraction of low-risk samples to get a balanced training dataset, as the dataset was biased towards high-risk cases. Possible-low-risk samples without any bad outcome were selected as candidates for the inclusion. Then, for each TMA, possible-low-risk samples were included/pseudo-labeled starting at longest follow-up time and descending until the TMA reached label balance or the source of possible-low-risk candidates was depleted.

**Fig. 4:**
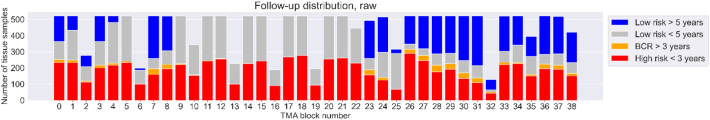
Label distribution over full dataset (train + test) for “raw” stage 1 labels. Each column is a TMA. Red: High risk, Blue: Low risk, Light gray: No bad outcome but follow-up shorter than 5 years, thus a “possible-low-risk”. Medium gray: Bad outcome, but not within 3 years.

**Fig. 5:**
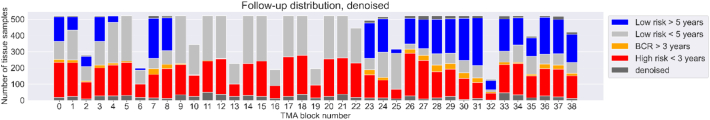
Label distribution over full dataset (train + test) showing denoised (discarded) samples in dark gray.

**Fig. 6:**
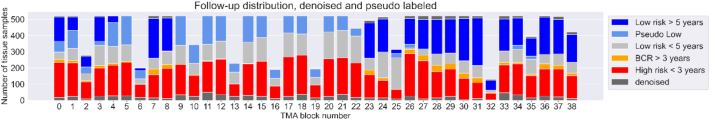
Label distribution over full dataset (train + test) showing also pseudo-labeled (included) likely low-risk samples in light blue.

Fig. 4, Fig. 5, Fig. 6 and Table 3 shows the different follow-up distribuitions in the different stages.

**Table 3:** Datasets prepared for stage 2 model.

Dataset	Number of samples	Fraction High risk	Fraction Low risk
Train set stage 1	8826	66.3%	33.7%
Test set stage 1 (raw testset stage 2)	1731	58.9%	41.1%
Train set denoised (not used)	7884	65.0%	35.0%
Test set denoised (cleaned testset stage 2)	1529	58.5%	41.5%
Train set denoised + pseudo labeled (train stage 2)	10 083	50.8%	49.2%

#### TMA-balancing

Some TMAs were quite unbalanced with regards to the binary risk division. So to reduce risk of training, the models to identify which TMA a sample had come from rather than identifying useful morphological risk features, a balanced set of low- and high-risk samples were randomly drafted from each TMA for the training set prior to each training epoch.

The pseudo-labeling synergized with this TMA-balancing in that it opened up to include also high-risk samples from those TMAs which had very low count of confirmed low-risk cases (over 5 years without BCR), and thus increased the variety in the dataset.

### Statistical methods

The ambition with our study has been to investigate if a model can be trained to predict prostate cancer outcome based on using objective outcome data in the simplest possible way just using the binary split between “good” and “bad” outcome. We were thus not aiming at creating a new survival analysis system as for instance presented in Ellery et al.^18^ However, the resulting continuous index seem to relate well to survival.

To evaluate our results, we used conventional statistical methods primarily AUC and balanced accuracy. We also calculated confusion matrices between our expert pathologists and our created PCAI indices. Additionally, we created Kaplan–Meier curves to show the survival times for patients stratified into groups based on our PCAI indices and for comparison ISUP groupings.

Confidence intervals for AUC are calculated with bootstrapping and *p*-test for comparison of 2 predictors AUC with permutation test, both methods calculated on 1000 iterations.

## Results

Test metrics was calculated against “bad outcome” as target. Both the uncurated “raw” stage 1 test set and the denoised (cleaned) test set were evaluated. The validity of the cleaned test set is discussed in section 4.4.

### Results on the “internal” test set

The internal test sets, i.e. the raw and cleaned test set drafted and held-out from the original training dataset, were used to evaluated performance of the PCAI score and compare this against case level ISUP. Note that the ISUP score has been graded having the full prostatectomy available and the PCAI score with only a 0.6 mm Ø tissue sample.

#### Raw test set metrics

Table 4 shows the PCAI and case level ISUP performance on the raw test set.

**Table 4:** Metrics on raw test set (tn=true negative, fp=false positive, fn=false negative, tp=true positive).

Model	AUC (95% CI)	Balanced accuracy	Confusion matrix [tn, fp, fn, tp]
PCAI	0.79 (0.77–0.81)	0.724	[514, 197, 109, 288]
Case level ISUP	0.81 (0.80–0.83)	0.753	[659, 52, 167, 230]

#### Cleaned test set metrics

Table 5 shows the PCAI and case level ISUP performance on the cleaned test set.

**Table 5:** Metrics on cleaned test set.

Model	AUC (95% CI)	Balanced accuracy	Confusion matrix [tn, fp, fn, tp]
PCAI	0.93 (0.92–0.94)	0.823	[513, 121, 56, 288]
Case level ISUP	0.84 (0.82–0.86)	0.782	[595, 39, 129, 215]

#### Receiver operating characteristics

**Fig. 7:**
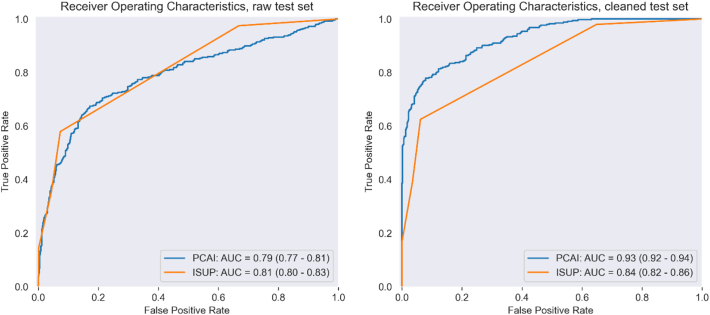
ROC curves for PCAI and ISUP respectively. Left column raw test set. Right column cleaned test set. 95% confidence interval within parentheses.

Receiver operating curves for the binary stratification case was calculated along with area under the curve (AUC) (see Fig. 7). For the raw test set, no statistical significant difference between the PCAI score and ISUP can be observed. For the cleaned test set the results are statistically significant (*p*<0.05).

#### Recurrence-free survival

**Fig. 8:**
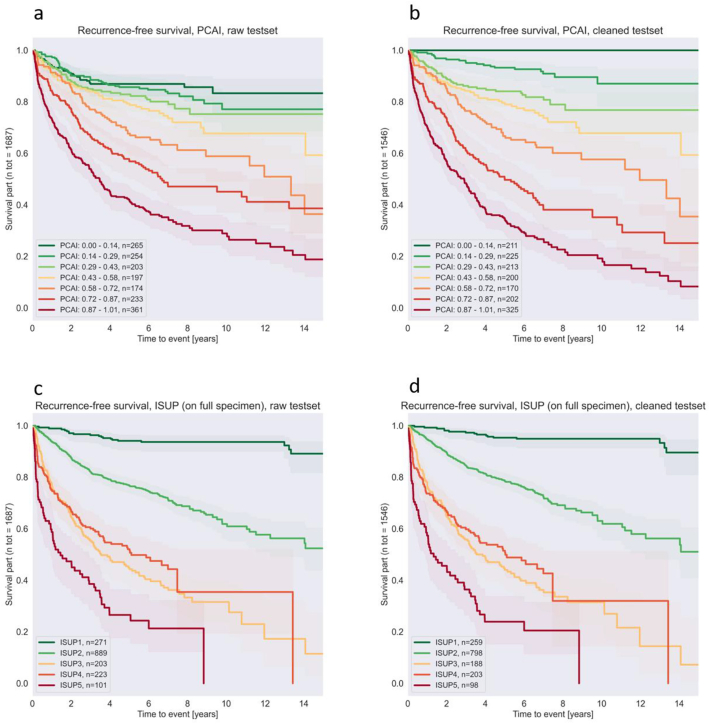
Recurrence-free survival for a–b: PCAI, c–d: Case level ISUP. Left column on raw test set. Right column on cleaned test set.

Kaplan–Meier curves were created to visualize risk stratification capability. The PCAI score is split into 7 groups. Whenever any case suffers from bad outcome, the curve drops. Thus, higher risk cohorts are expected to have a curve more drastically dropping than lower risk cohorts (see Fig. 8).

Unadjusted and adjusted Cox proportional hazard ratios were calculated for PCAI and ISUP (see Table 6).

**Table 6:** Cox proportional hazard ratios. The adjusted ratios were adjusted for age and PSA value.

Risk factor	Test set	Hazard ratio (95% CI)	*p*-value	Concordance index
PCAI	Raw	1.28 (1.24–1.32)	<0.005	0.70
PCAI, adjusted	Raw	1.25 (1.22–1.29)	<0.005	–
ISUP	Raw	1.38 (1.33–1.42)	<0.005	0.70
ISUP, adjusted	Raw	1.34 (1.29–1.39)	<0.005	–
PCAI	Cleaned	1.47 (1.42–1.53)	<0.005	0.78
PCAI, adjusted	Cleaned	1.44 (1.39–1.50)	<0.005	–
ISUP	Cleaned	1.41 (1.36–1.46)	<0.005	0.72
ISUP, adjusted	Cleaned	1.37 (1.32–1.42)	<0.005	–

For PCAI, the hazard ratios were calculated for 0.1 units of change, for case level ISUP 0.5 units of change. Giving both a range of 0–10.

### Comparison against expert pathologists

The Test-UKEHDS test set consists of 4181 TMA spots on 828 cases with an average of 5 spots per case which had all been Gleason-annotated by 2 expert uropathologists. The spots were all individually analyzed and then the maximum score was chosen to represent the case score, from which the statistics were calculated.

#### Test-UKEHDS

**Fig. 9:**
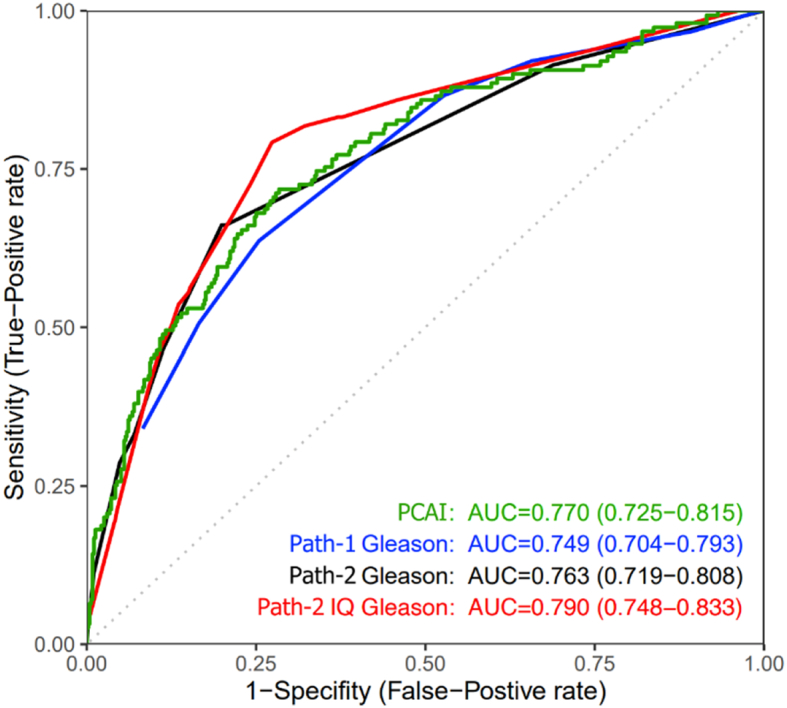
ROC curves showing performance of our 2 expert pathologists using standard Gleason, Pathologist 2 also using the extended IQ Gleason, and our PCAI index. Path-1 IQ Gleason vs. Path-2 Gleason *p* < 0.05, others *p* > 0.05. IQ-Gleason differs from the standard Gleason score in that it takes the relative amount of high-grade into consideration which gives a score with higher resolution and proven better correlation with outcome – which is also visible here.^19^ 95% confidence interval within parentheses.

The result from this test shows that PCAI have a higher AUC than both Pathologist 1 and Pathologist 2 on “standard” Gleason but gets slightly below Pathologist 2 IQ-Gleason. The differences in AUC between PCAI and the pathologists are however small and not statistically significant (*p* > 0.05) (see Fig. 9).

To compare score similarity between the annotators and PCAI, a weighted kappa score (κ) was calculated on the confusion matrices with the PCAI score quantified in 5 segments, as a metric of agreement (higher kappa means higher agreement).

The weights for the kappa calculation were set as the distance in blocks in the confusion matrix divided by the maximum distance in terms of number of blocks.

Highest agreement per the kappa score was given between PCAI and Pathologist 1. However, the fact that the comparison is done between different metrics (Gleason is ordinal where PCAI is continuous) limits the value of this comparison. It is also important to be aware that neither the Gleason grades nor the PCAI grade represents the ground truth. But the comparison shows that it is much more common that PCAI predicts worse outcome than what the subjective grading does than the other way around. Also, only a small fraction of cases in the lower half of the PCAI scale receive Gleason scores at 4+3 or higher.

The confusion matrices are shown in Table 7, Table 8, Table 9.

**Table 7:** Confusion matrix between Pathologist 1 Gleason annotations and PCAI scores.

		*Pathologist 1*							
		3+3	3+4	4+3	4+4	4+5, 3+5	5+4, 5+3	5+5	*N*
*PCAI*	0.0–0.2	558	137	43	32	0	1	1	772
	0.2–0.4	447	223	74	92	9	4	1	850
	0.4–0.6	234	160	72	99	22	9	9	605
	0.6–0.8	96	86	67	155	31	38	17	490
	0.8–1.0	26	36	59	256	99	125	162	763
	*N*	1361	642	315	634	161	177	190	3480

κ_PCAI_p1_ = 0.35

**Table 8:** Confusion matrix between Pathologist 2 Gleason annotations and PCAI scores.

		*Pathologist 2*							
		3+3	3+4	4+3	4+4	4+5, 3+5	5+4, 5+3	5+5	*N*
*PCAI*	0.0–0.2	524	148	4	0	1	0	0	677
	0.2–0.4	451	250	12	7	2	0	0	722
	0.4–0.6	232	259	27	6	1	2	0	527
	0.6–0.8	112	232	44	24	16	3	4	435
	0.8–1.0	74	237	141	95	90	45	53	735
	*N*	1393	1126	228	132	110	50	57	3096

κ_PCAI_p2_ = 0.20

**Table 9:** Confusion matrix between Pathologist 1 and Pathologist 2. Here spots are aggregated per case.

		*Pathologist 1*							
		3+3	3+4	4+3	4+4	4+5, 3+5	5+4, 5+3	5+5	*N*
*Pathologist 2*	3+3	52	77	23	37	9	4	2	204
	3+4	18	65	67	118	51	18	14	351
	4+3	1	2	10	34	17	17	18	99
	4+4	0	1	0	18	9	11	16	55
	4+5, 3+5	1	1	0	2	4	18	22	48
	5+4, 5+3	0	0	0	1	1	5	18	25
	5+5	0	0	0	0	1	4	21	26
	*N*	72	146	100	210	92	77	111	808

Κ_p1_p2_ = 0.29

### Test-CB

Additionally, 500 randomly chosen samples (Test-CB) were annotated by Pathologist 1. These images were chosen purely at random and was not cleaned of non-representative samples (no epithelium tissue, image artifacts, etc). Therefore, in addition to annotating a cancer risk level on a scale from 1 to 5 (like ISUP), the pathologist also annotated an assessability score of 0 to 1 and the reason if a sample was not assessable. The samples not considered assessable by the pathologist were not included in the comparison between AI and pathologist as no confident grading could be established by the pathologist on those.

**Fig. 10:**
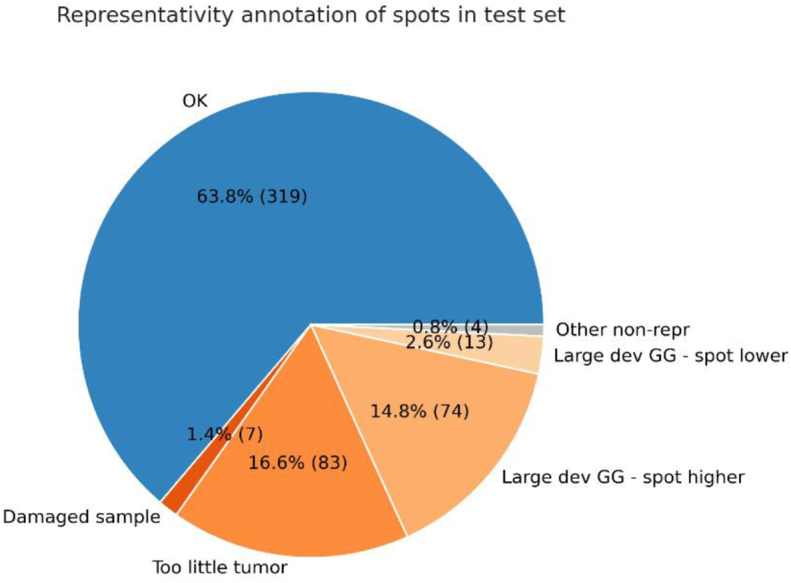
Representativity of the spots with regards to sample quality and case-level (full prostatectomy) ISUP (GG) vs Pathologist TMA-spot risk grading.

By this annotation data, the representativity of the spots could be analyzed, as shown in Fig. 10. As can be seen from the figure, the small area available in the spots have some problems in representing the case. This need to be taken into account when evaluating the outcome of the tests.

Further in the metrics – some more had to be removed from calculating metrics due to no definable risk group according to PCAI risk definition. After removing those images and those not deemed assessable. 400 samples remained for survival analysis and 201 with a definable binary risk group. All 500 samples were withheld from the training set.

The samples in the Test-CB test set were annotated by Pathologist 1 given the task to perform a subjective risk grading in a scale 0–5 with the threshold between low- and high risk set between 2 and 3.

#### Raw test set metrics

Table 10 shows the PCAI, Pathologist 1, and case level ISUP performance on the raw Test-CB test set.

**Table 10:** Results from PCAI, Pathologist 1 and case level ISUP on the raw/non-cleaned Test-CB test set.

Model	AUC (95% CI)	Balanced accuracy	Confusion matrix [tn, fp, fn, tp]
PCAI	0.77 (0.70–0.82)	0.716	[76, 36, 22, 67]
Pathologist 1	0.73 (0.67–0.79)	0.695	[79, 33, 28, 61]
Case level ISUP	0.89 (0.86–0.92)	0.793	[106, 6, 32, 57]

Case level ISUP is statistically significantly higher than both PCAI and Pathologist 1 (*p* < 0.05). No statistically significant difference can be observed between PCAI and Pathologist 1 (*p* > 0.05).

#### Cleaned test set metrics

Table 11 shows the PCA, Pathologist 1, and case level ISUP performance on the cleaned Test-CB test set.

**Table 11:** Results from PCAI, Pathologist 1 and case level ISUP on the cleaned Test-CB test set.

Predictor	AUC (95% CI)	Balanced accuracy	Confusion matrix [tn, fp, fn, tp]
PCAI	0.93 (0.90–0.96)	0.833	[76, 21, 9, 67]
Pathologist 1	0.84 (0.78–0.88)	0.793	[76, 21, 15, 61]
Case level ISUP	0.92 (0.89–0.95)	0.833	[94, 3, 23, 53]

**Fig. 11:**
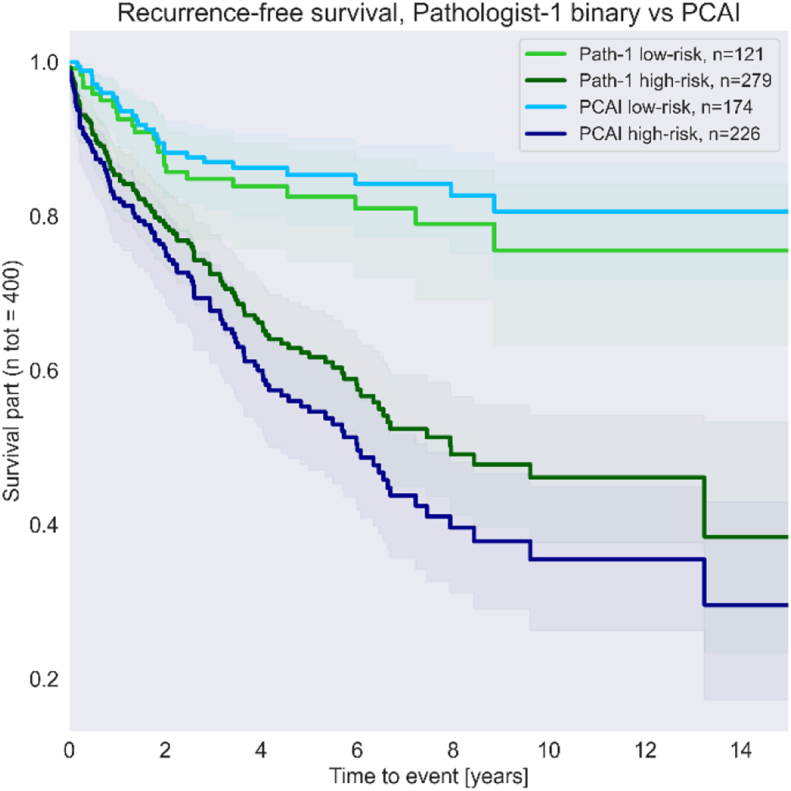
Recurrence-free survival. PCAI vs Pathologist 1 samples grouped into low- and high-risk cohorts. Larger separation between high- and low-risk cohorts are better. (Raw test set).

**Fig. 12:**
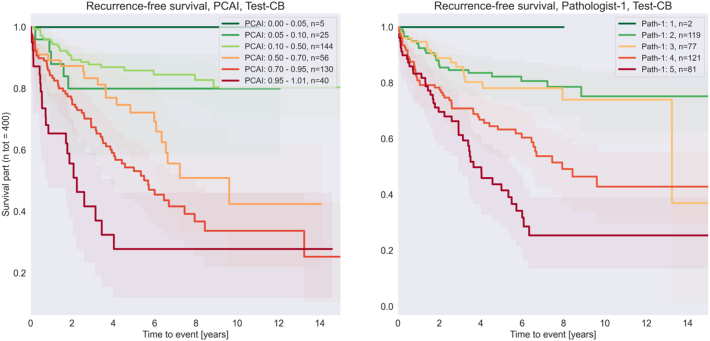
Recurrence-free survival on the Test-CB test set. Left: PCAI, Right: Pathologist 1. (Raw test set).

No statistically significant difference can be observed between Case level ISUP, PCAI and Pathologist 1 (*p* > 0.05) (see Fig. 11, Fig. 12).

### Effect of balanced training

To investigate the effects of the balanced selection of samples, 3 different models were trained with exactly the same architecture, but different training set selection.

- Global balancing selection: Samples from the training set were drafted before each epoch in a balanced 50/50 high/low risk distribution, without considering TMA balance.
- TMA-balanced selection: Samples from the training set were drafted before each epoch, from each TMA in a balanced 50/50 high/low risk distribution.
- TMA-balanced selection on denoised and pseudo-labeled test set: Samples from the training set were drafted before each epoch, from each TMA in a balanced 50/50 high/low risk distribution. Additionally likely non-representative samples were discarded and some likely low-risk samples were included to improve TMA-balance.

Rationale (why?):

- TMA-balancing: To balance out and reduce risk for overfit against TMA-specifics such as staining and slice thickness.
- Denoising: To train model on a more representative training set.
- Pseudo-labeling: To increase training set size. Including some likely low-risk samples allows the inclusion of confirmed high-risk samples from TMAs with shorter follow-up than 5 years which otherwise would have been blocked for train set drafting with TMA-balanced draft.

The model was the PCAI architecture with scale 0.5 (20x) and 352 px patchsize.

The evaluation was performed on the same test samples and “bad outcome” as endpoint (no pseudo-label inclusion).

Table 12 shows performance metrics on the 3 models trained with the different sampling procedures.

**Table 12:** The effect of different sampling procedures on the performance of the model.

Model	AUC	AUC	Train loss (approx)	Val loss (approx)
	Raw test set	Cleaned test set		
Trained on global balancing selection, raw train set	0,769	0,871	0.52	0.61
Trained on TMA-balanced sample selection, raw train set	0,777	0,908	0.60	0.58
Trained on TMA-balanced sample selection, denoised and pseudo-labeled train set	0,780	0,917	0.42	0.45

The performance increase shows that the training scheme worked as intended. The performance increased between TMA-balancing and global balancing which together with the train and validation loss suggests that the TMA balancing reduces overfit compared to the global balancing scheme.

### The cleaned test set – is it valid?

The denoising was performed to discard unrepresentative samples. This is quite safe to do on the training set. However – is this cleaning valid on the test set?

The obvious risk is that the first cleaner-model at stage 1 (cleaning stage) is quite similar to the final stage 2 model and that the removed samples are ones that the cleaner model had a hard time with and couldn’t correctly predict rather than unrepresentative samples. Then if the models are similar the stage 2 model will benefit in testing from having hard cases removed and the metrics will be falsely high.

But, as the TMA spots are so small, only 0.6 mm diameter, there is a non-negligible risk for the spots being sampled at a non-representative location, i.e. the spot is unrepresentative to the whole prostate. This is illustrated by the study of a subset of the spots described in section 4.3.

The spots might be representative to the whole prostate, but the outcome label might not be representative to the risk of the cancer. I.e. patients who had high-risk factors but did survive anyway by “fortune” or by being cured, and “unfortunate” low-risk patient that had bad outcome anyway.

How to test then?

• If the denoising removes unrepresentative spots with regards to the whole prostate, the spot level metrics should increase but not affect case level metrics.
  o Different spot level predictors (PCAI and annotator) should experience about the same increase.
• If the denoising removes unrepresentative labels with regards to the case, all predictor metrics should increase.

Results from the test metrics for the raw and cleaned test sets are shown in Table 13:

**Table 13:** The effect of different testing procedures on the overall performance of the model.

Predictor	Test set	Input context	AUC	AUC	AUC change
			Raw testset	Cleaned testset	Raw -> Cleaned
PCAI	Test-CB	Spot	0,765	0,935	0.17 (+22% rel)
Pathologist 1	Test-CB	Spot	0,729	0,835	0.11 (+15% rel)
Case level ISUP	Test-CB	Case	0,889	0,922	0.03 (+4% rel)

PCAI	Test	Spot	0,789	0,929	0.14 (+18% rel)
Case level ISUP	Test	Case	0,815	0,842	0.03 (+3% rel)

Pathologist 1 metrics can be seen as the result of expert ISUP annotation on spot level, and the Case level annotation as expert ISUP annotation on case level. They are therefore measuring the same quantity but on different scales (spot vs whole prostatectomy). The pathologist’s AUC was increased by 15% and case level by 4%. This argues that spots with unrepresentative sampling location were removed.

The case level metrics rose somewhat with the cleaning which argues that to some degree spots with unrepresentative cases were removed.

The PCAI metrics was raised more than the pathologist’s metrics which argues that to some degree the denoising made the case “easier” for the PCAI model.

The cleaned test metrics can thus not safely be used to represent the “true” metrics. But neither the raw test metrics. The “true” metrics lie somewhere in between.

## Discussion and conclusion

### PCAI performance

PCAI gets higher AUC than the expert pathologists in both the smaller Test-CB test set and the larger Test-UKEHDS test set, when the pathologists are doing standard Gleason. Pathologist 2, however, achieves the best performance when doing IQ-Gleason. The differences are however small and are not statistically significant in any of the cases.

PCAI is clearly doing an analysis in class with expert uropathologists in ability to predict bad outcome for prostate cancer patients. It cannot however be proved here that PCAI goes beyond the systematic limit set by Gleason’s gland-centered analysis, even if it is reasonable that it could do so due to it having access to a richer set of information.

An aggeregation of the reusults for comparison of the different predictors are shown in Table 14

**Table 14:** The performance of different predictors in terms of the AUC measure. This table is a collection of previously presented metrics from the results section.

Predictor	Test set	AUC (95% CI)
PCAI	Test-UKEHDS	0.77 (0.73–0.82)
Pathologist 1, Standard Gleason	Test-UKEHDS	0.75 (0.70–0.79)
Pathologist 2, Standard Gleason	Test-UKEHDS	0.76 (0.72–0.81)
Pathologist 2, IQ-Gleason	Test-UKEHDS	0.79 (0.75–0.83)

PCAI	Test-CB, raw test set	0.77 (0.70–0.82)
Pathologist 1	Test-CB, raw test set	0.73 (0.67–0.79)

PCAI	Test-CB, cleaned test set	0.93 (0.90–0.96)
Pathologist 1	Test-CB, cleaned test set	0.84 (0.78–0.88)

### Conclusion

We have shown that it is possible to train an AI to predict the outcome of prostate cancer based on image analysis of tissue samples without any subjective human input in the process. The resulting prediction accuracy is comparable to what can be achieved by an expert pathologist using the established Gleason grading system.

Our study was based on relatively small selected tissue samples from each patient. For useful clinical application a grading system must work with tissue samples obtained through needle biopsies. Such samples typically include up to 100 times more tissue than we have had available in our TMA spots. Most of that tissue will for most patients be normal not cancerous. This could potentially present a problem for our AI model. We have developed a cancer detector that shows similar performance to those described in Bulten et al,^10^ that is with 0.98 AUC in discriminating between normal and cancerous tissue. After application of that detector, only tissue areas showing cancer will be subject to analysis by the PCAI grading algorithm. Preliminary results show that the grading through this 2-stage process works well. Our system is able to show which parts of the tissue sample was most important for the decision increasing the trustworthiness of the result.

These results need to be verified on biopsies in large-scale studies on independent material from more than one clinic and could if it stands the test become the basis of a new more objective and reproducible way of grading prostate cancer in preparation for deciding patient treatment strategies.

### Further work

Our study was based on samples prepared and scanned with the same scanner in the same laboratory, although over a relatively long time period. We have performed some preliminary studies indicating that we get similar results when doing changes to the preparation procedures and using different scanners. But much more extensive such studies are needed to show the stability of the developed PCAI grading algorithm.

To further understand the systematic limit of the Gleason system and the individual contribution of, and interaction between, information from cells, stroma, gland structure, and possibly other categories, we have designed a study to isolate those individual features and study how far AI models can go by having only those features available, and combinations of them, in training and prediction.

Further, it is of interest to establish a test set or test method which tells the “true” performance as close as possible and makes it possible to separate true performance from test set label noise (unrepresentative samples etc.).

## Ethical approval

The use of archived remnants of diagnostic tissues for manufacturing of TMAs and their analysis for research purposes as well as patient data analysis has been approved by local laws (HmbKHG, §12) and by the local ethics committee (Ethics commission Hamburg, WF-049/09).

All work has been carried out in compliance with the Helsinki Declaration.

## Conflict of interest

Walhagen, Bengtsson and Busch are shareholders in Spearpoint Analytics AB a company developing AI and digital pathology solutions.
